# Capsid protein is central to the birth of flavivirus particles

**DOI:** 10.1371/journal.ppat.1008542

**Published:** 2020-05-28

**Authors:** Ter Yong Tan, Guntur Fibriansah, Shee-Mei Lok

**Affiliations:** 1 Programme in Emerging Infectious Diseases, Duke–National University of Singapore Medical School, Singapore, Singapore; 2 Centre for BioImaging Sciences, Department of Biological Sciences, National University of Singapore, Singapore, Singapore; Mount Sinai School of Medicine, UNITED STATES

## Overview of flavivirus infection cycle

**Fig 1:**
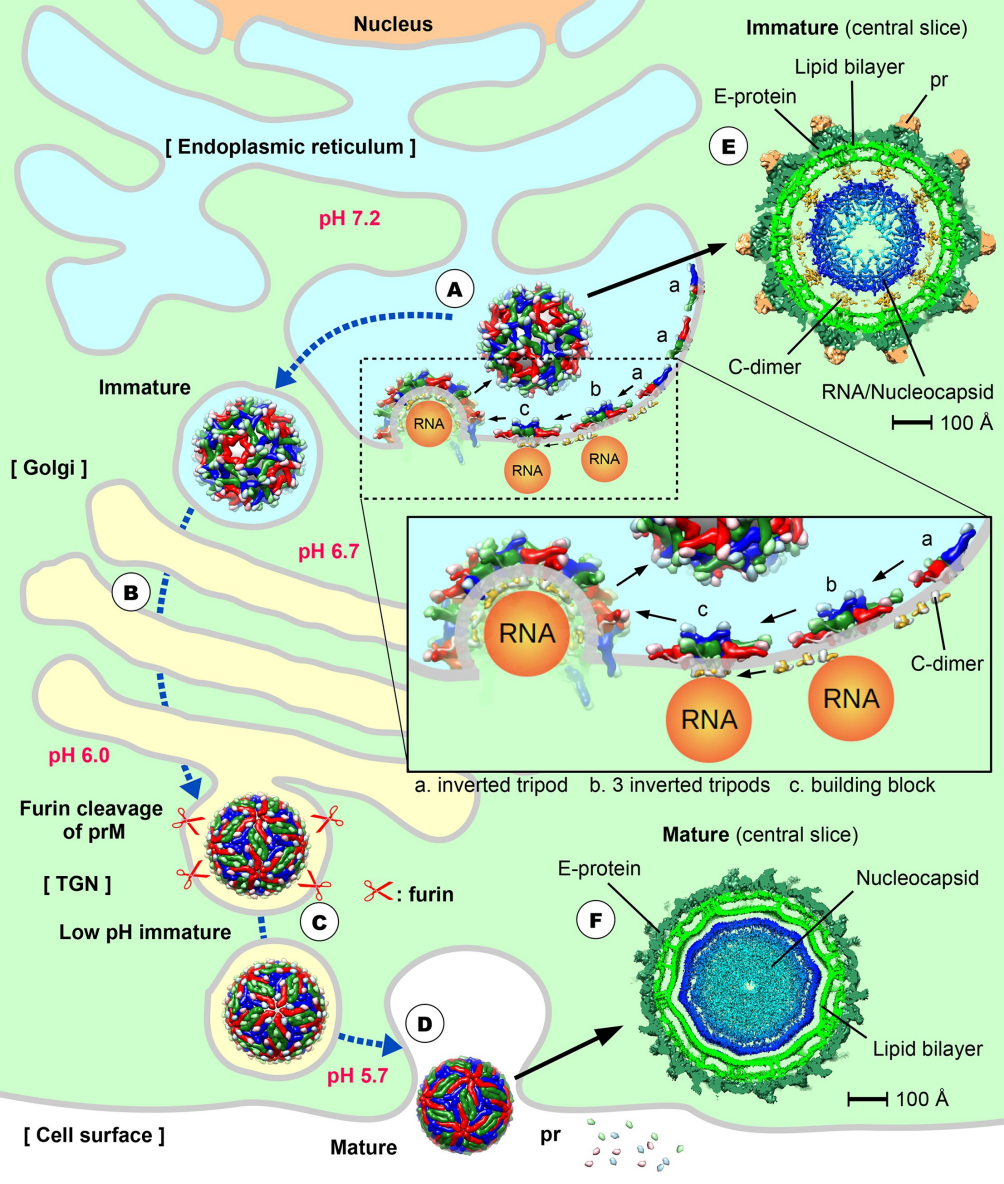
A schematic diagram of the flavivirus maturation pathway. (A) The viral RNA is translated on ER to produce polypeptide. This polypeptide is digested by host peptidase and viral proteases to form 10 flavivirus proteins, including the structural proteins: E, prM, and C. In the lumen side of ER, E and prM form a heterodimer, and 3 of these heterodimers interact with each other, forming an inverted tripod structure. The tripods then interact with each other. On the cytoplasm side, a C dimer binds to the TM regions of a prM–E tripod and also the viral RNA genome. One C dimer then interacts with 2 other nearby C dimers via their α5 helices to form a triangular network. The end result is one side of the C dimers interacts with lipid bilayer/TM regions of prM–E, while the other interacts with RNA. The flavivirus particle is assembled on the ER membrane, and it then buds off as spiky immature particles. (B) The immature particles are transported through the TGN. A conformational change of the virion from spiky to smooth surface morphology is triggered by the exposure to the more acidic pH environment of the TGN. The virus surface conformational changes expose the furin-cleavage site on the prM, allowing the prM to be cleaved into pr and M proteins. (C) The cleaved pr molecule remains attached to the viral particles at the low pH environment of the exosomes. (D) When released into the extracellular environment, the increased in pH causes the release of pr molecules from the virus surface, forming the fully infectious virus particle. (E) The central slice of the cryoEM density map of immature particles, showing the C dimers bridging the inner leaflet of the lipid bilayer and the RNA genome. (F) The center slice of the cryoEM density map of the mature virus particles, showing a lack of C dimer density at the interface between the inner leaflet of the bilipid layer membrane and the RNA genome. We speculate that the dramatic rearrangement of the E and prM/M proteins during the virus maturation process may result in the release of the C dimers from the inner leaflet of lipid bilayer membrane. The approximate luminal pH values of the cellular compartments are shown [28]. C, Capsid; cryoEM, cryo-electron microscopy; E, Envelope; ER, endoplasmic reticulum; prM, precursor Membrane; TGN, trans-Golgi network; TM, transmembrane.

**Fig 2:**
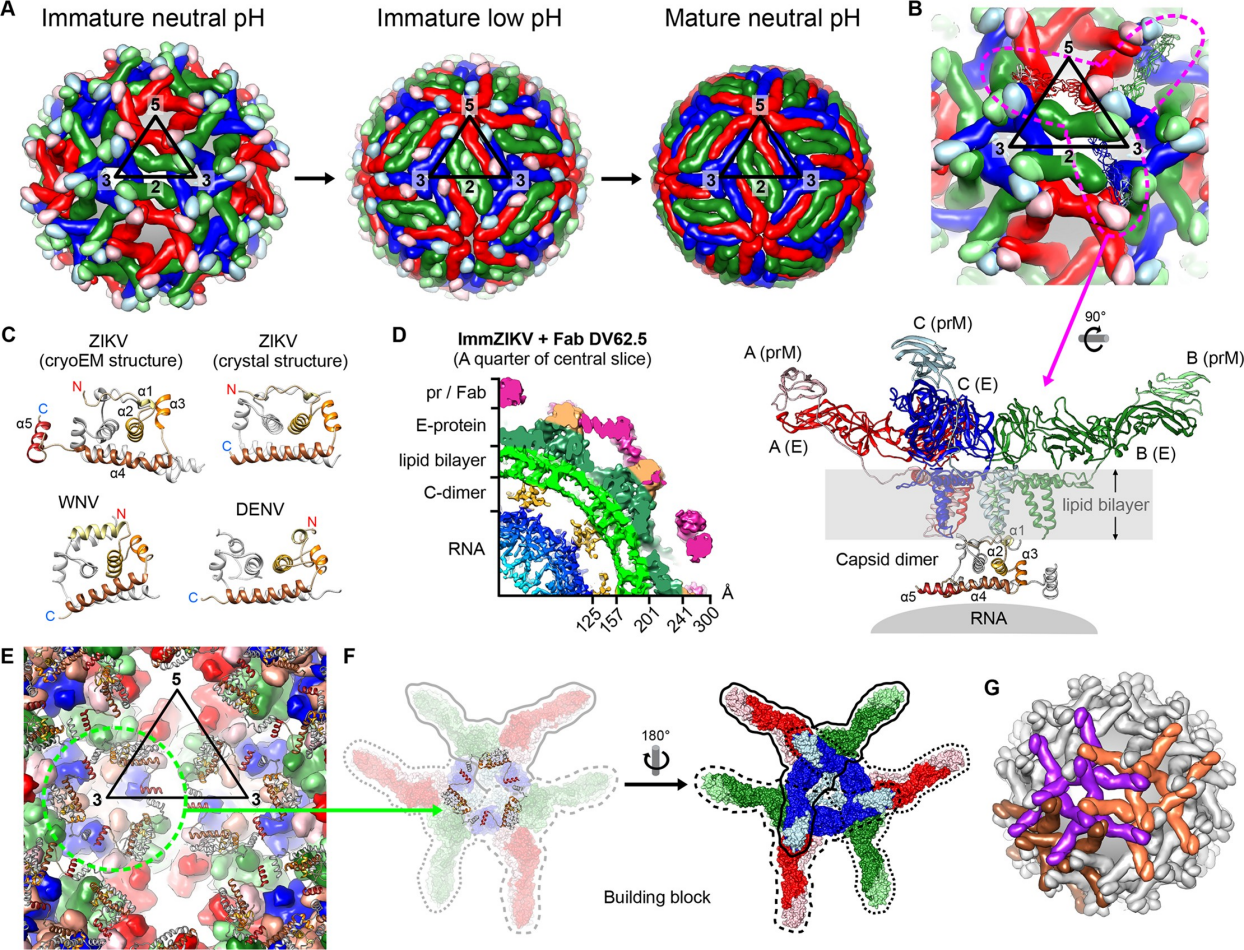
The organization of structural proteins in flavivirus. (A) E protein or prM–E protein arrangement on immature (left), low pH immature (center), and mature flavivirus (right) during the maturation process. An icosahedaral asu is shown as a black triangle with 5-, 3-, and 2-fold vertices indicated. The 3 individual E proteins in an asu are each located near 5-, 2-, or 3-fold vertices and are colored in red, green, and blue, respectively. (B) Three prM–E heterodimers form an inverted tripod. One of the inverted tripods is indicated with a dashed magenta line. The inverted tripods crisscross with other neighboring inverted tripods, forming the spiky immature virus surface. Underneath an inverted tripod and interacting with the TM regions of 3 E–prM is a C protein dimer. One of the C protein protomers within a dimer is colored with a gradient of brown shades, starting with lighter shades at the N-terminal end. The other protomer is colored in gray. The helices α1–α5 on the colored C protomer is indicated. (C) Comparison of the structures of flavivirus C proteins [8–11]. The N-terminus (red N) and C-terminus (blue C) of the C proteins are shown. (D) A quarter of the central slice of Fab DV62.5 complexed immature ZIKV cryoEM map. (E) Inside-out view of the ImmZIKV structure showing the C protein dimer organization in the immature particle around the 3-fold vertex (green dashed circle). One C dimer formed a triangular network with the other 2 C dimers through the interactions between the α5 helices. (F) The basic building block for the virus shell consists of a triangular network of 3 C dimers interacting with 9 E–prM heterdimers. (G) One building block interacts with another through the tips of their E–prM heterodimers on the outside of the particle, thus forming a lattice. Three neighboring building blocks are shown and colored in orange, purple, and brown. asu, asymmetric unit; C, Capsid; cryoEM, cryo-electron microscopy; E, Envelope; Fab, antigen-binding fragment; ImmZIKV, immature ZIKV; prM, precursor Membrane; ZIKV, Zika virus.

Flaviviruses such as dengue virus (DENV) and Zika virus (ZIKV) are enveloped, positive-sense single-stranded RNA viruses. Upon entry into a host cell by receptor-mediated endocytosis, the virus particle undergoes low-pH–driven endosomal membrane fusion to release its genome into the cytoplasm [1]. This viral genome is then translated into a single polyprotein that is co- and post-translationally processed into 3 structural and 7 nonstructural proteins. The 3 structural proteins that form the immature virus particle are the Capsid (C), Envelope (E), and precursor Membrane (prM) proteins. After formation of the core, consisting of the C protein complexed with the newly synthesized viral RNA genome, the core then buds into the endoplasmic reticulum (ER) membrane decorated with membrane-anchored viral E and prM proteins, forming an immature virus (Fig 1A). The immature virion then undergoes maturation during transport through the secretory pathway (Fig 1B). This involves large conformational rearrangements of the E proteins (Fig 2A), which enhances the cleavage of the prM to M protein by furin proteases (Fig 1C) to generate the infectious mature virus particle (Fig 1D).

## The surface protein structures prM and E of the immature and mature flavivirus particles

Cryo-electron microscopy (cryoEM) structures of the immature and mature flavivirus show that their surface proteins, although they are organized in a vastly different way, both exhibit icosahedral symmetries (Fig 2A) [2–4]. These virus surfaces are made up of 180 copies each of the E and prM/M (prM in immature and M in mature) proteins [2]. On the surface of the immature virus in the neutral pH environment of the ER where they are first assembled, they contained 60 spikes (Fig 2A, leftmost). This surface architecture is made from an assembly of 60 inverted tripods that crisscross with each other. Each of these inverted tripods consists of 3 prM–E heterocomplexes (Fig 2B). The base of the inverted tripod (Fig 2B) is anchored to the underlying lipid membrane by their transmembrane (TM) helices. Each of the ectodomains of these 3 prM–E complexes then interacts with those from other neighboring tripods, imparting a spiky appearance to the immature virus (Fig 2A, leftmost).

During the egress of the immature virus through the acidic compartments of the trans-Golgi network, the low pH triggers the reorganization of the heterotrimeric spikes into a dimeric surface protein organization (Figs 1A–1C and 2A) [5]. This dramatic structural rearrangement of the surface glycoprotein is a prerequisite for exposing the furin-cleavage site on the prM for processing by the furin protease (Fig 1C). Once the prM is cleaved, the pr peptide remains bound to the virus at an acidic pH and only detaches upon excretion of the particle into the neutral pH extracellular environment (Fig 1C and 1D) [5]. The final infectious mature flavivirus particles have a smooth spherical appearance, and the E proteins exist as homodimers lying flat on the surface of the virus lipid membrane (Figs 1D, 1F and 2A) [2, 6, 7]. Three such E protein dimers are laying parallel to each other, forming a raft, and 30 such rafts are arranged in a herringbone pattern (Fig 2A, rightmost).

## Flavivirus C proteins are instrumental in gluing the virus RNA genome and surface proteins together for the formation of infectious virus particle

Although the virus assembly process is a crucial stage in the virus infection cycle (Fig 1A), its molecular mechanism is poorly understood. C protein, the first flaviviral protein being translated, plays a central role in interacting with the viral RNA genome for packaging into the virus particle during the assembly process. However, how the C protein–RNA complex can associate with the ER lipid membrane that has prM/E proteins anchored on it for virus budding is largely unknown.

While translating the virus RNA genome, the synthesized viral polyprotein is threaded back and forth through the ER membrane; while some of the viral proteins are facing the cytoplasm, the others face the lumen of the ER. The full-length C (f-C) protein, at this point of processing, is largely facing the cytoplasm. The f-C protein contains a disordered N-terminus followed by 5 α-helices (helices α1–α5). The f-C protein helix α5 is a short hydrophobic helix that immediately precedes the prM in the polyprotein, and it helps the prM traverse the ER membrane so as to bring the prM into the ER lumen. The N- and C-terminal ends of this helix α5 in the ER membrane contain cleavage sites for viral NS2B/NS3 protease and host signal peptidase, respectively. When fully processed by these 2 proteases, the end result is the mature C (m-C) protein containing helices α1–α4 in the cytoplasmic side of the ER, leaving behind the helix α5 in the ER membrane, and the prM protein in the ER lumen.

NMR and X-ray crystal structures of the flavivirus m-C dimer show there is structural conservation among the different flaviviruses despite their poor sequence similarity [8–11] (Fig 2C). The 4 α-helices of each protomer are held together by short loops, and they form a 3-layer structure. There is an asymmetric charge distribution on the C dimeric surface: one side contains hydrophobic patches that are mainly contributed by helices α2, while the opposite side contains positively charged residues of helices α4. This leads to the postulation that the hydrophobic residues on one side likely interact with the viral lipid membrane, while the α4 helices on the opposite side interact with the negatively charged RNA genome [10].

Unlike other flaviviruses, the ZIKV m-C protein has a long pre-α1 loop that precedes a short helix α1 [11]. The helix α1 in ZIKV is shorter compared to the other flaviviruses because there are 2 proline residues, thus breaking the helix. The resulting long pre-α1 loop extends the dimerization surface and enhances interactions between the 2 m-C protomers [11]. The orientation of helix α1 with respect to helix α2 can affect the accessibility of the hydrophobic residues on helix α2. Interestingly, the helix α1 of neuropathic flaviviruses such as ZIKV and West Nile virus (WNV) is positioned perpendicular to helix α2 in a “close conformation” [12]. Non-neuropathic flavivirus like DENV have helix α1 lying parallel to helix α2 in an “open conformation” [10]. The open conformation allows more hydrophobic residues from helix α2 to be more accessible; however, its functional significance is unknown.

Even though the cryoEM structures of surface proteins on mature and immature flavivirus particles (WNV, DENV, ZIKV, and yellow fever virus) have been determined, some to near-atomic and subnanometer resolutions, the densities of the C proteins inside these virus particles are either poor or absent. The first glimpse of a low-resolution density, sandwiched between the core and viral lipid membrane, that somewhat correlates to the volume of the C protein dimer was reported in the immature ZIKV (ImmZIKV) by Prasad and colleagues [13]. This suggests that ImmZIKV could have a more stable C protein structure. Our recent paper, Tan and colleagues [14], uses an antigen-binding fragment (Fab) of HMAb DV62.5, which binds across the prM and E proteins, to stabilize the ImmZIKV surface, which in turn stabilizes the entire virus particle, allows us to observe the C dimeric protein density to 9-Å resolution (Fig 2D). We then fitted the crystal structure of ZIKV m-C protein into this density and showed that the C dimers interact with both the RNA core and the E–prM TM region (Fig 2B). This suggests the C dimer may be the factor that ensures all virus particle are packaged with the RNA genome.

We also observed in the cryoEM density map that some of the C proteins inside the virus particle are actually that of the f-C protein [15], showing clear density of the helix α5, although it is slightly weaker compared to the rest of the C protein. Consistent with the cryoEM density map, mass spectrometry and SDS-PAGE of purified virus sample showed a mixture of m-C and f-C proteins. We therefore fitted helix α5 into its corresponding density at the C-terminal end of the fitted m-C crystal structure and showed that helix α5 plays a big role in the organization of the overall quaternary ImmZIKV structure. The neighboring α5 helices from adjacent f-C dimers interact with each other via hydrophobic interactions to form trimers of f-C dimers (Fig 2E).

The results showed the basic building block that makes up the final architecture of the virus particle. They are the trimers of a mixture of f- or m-C dimers complexed with 9 preformed prM–E proteins (Fig 2F). The ectodomains of prM–E complexes of these basic building blocks then interact with each other on the lumen side of the ER, assembling into an icosahedral virus particle (Fig 2G).

## Can flavivirus assemble in the absence of C protein?

Coexpression of only the prM and E from flaviviruses such as tick-borne encephalitis virus and DENV showed that these proteins can rapidly assemble into empty subviral particles, and the organization of these proteins are very different from that of the infectious virus particles [16]. The subviral particles showed different sizes, and the most abundant form contains only 60 prM/E proteins on its surface, in contrast to the 180 copies of prM/E on the infectious virus particles [16]. This observation suggests that lateral interactions between prM–E complexes could somewhat partially drive virus assembly, but additional factors are needed for the formation of the correct infectious virus architecture.

## What happens to the C protein within the virus particle during flavivirus maturation?

Unlike the immature virus (Fig 1E), cryoEM density maps of the mature form of flavivirus do not show any C protein density (Fig 1F). Previously, we have determined the structural rearrangements of the surface proteins prM and E during the DENV maturation process, and it shows that the cluster of 3 prM and E TM regions in the immature virus reorganized into a dimeric structure in the mature virion [2]. In our current paper, since a C protein dimer interacts simultaneously with the cluster of E/prM TM regions in the immature virus, the dramatic reorganization of these TM regions during maturation may cause C protein to dislodge from prM and E proteins. We speculate that this may cause the C-RNA to condense to the inside the core of the mature virus, thus allowing the release of the C-RNA core into the cytoplasm after the virus and endosomal membrane have fused in the next cycle of infection.

## Comparison of the flavivirus assembly process with other viruses

Alphaviruses are small, enveloped, positive-sense single-stranded RNA arboviruses that are closely related to flavivirus. CryoEM structures of these viruses show that both follow icosahedral symmetry, although alphavirus has a T = 4 whereas flavivirus has T = 3 symmetry. They have structurally similar surface glycoproteins—E1 in alphavirus and E protein in flavivirus—and an inner C protein layer that surrounds an RNA core. However, their assembly processes are very different—alphavirus is assembled on the plasma membrane with a preassembled nucleocapsid core (RNA–C protein complex), while in flavivirus, virus assembles in the ER in a concerted manner along with the translation of the polypeptide chains [14, 17–19]. It is possible that the flavivirus assembly process may somewhat be more applicable to other envelope viruses that bud off from the ER. One such virus is hepatitis B virus (HBV); however, empty particles with no DNA genome form the majority of the particles released from the cell, which indicates that its DNA-binding protein (equivalent with C protein in flavivirus) may not engage directly with its surface protein, and hence, its assembly process is likely very different [20]. Coronavirus, an RNA envelope virus, also assembles in the ER, with the binding of surface protein M to the N protein–RNA complex packaging the genome into the viral particle [21]. However, the viral particle is pleomorphic (not organized in icosahedral symmetry), suggesting that there is no single assembly unit (a brick in the house) to form the final virus architecture, unlike flaviviruses [22]. This shows that the mechanism of virus assembly process can be quite diverse.

## Can we therapeutically target C protein to interfere with flavivirus assembly?

The feasibility of targeting C protein has been demonstrated in a number of viruses such as HBV and HIV and even flaviviruses such as DENV [23, 24]. The small-molecule drug ST-148, for instance, has been shown to induce DENV C protein self-association/aggregation [25, 26]. This effect of ST-148 is thought to interfere with virus nucleocapsid assembly, resulting in a broad antiviral activity against all serotypes of DENV that is seen both in vitro and in vivo. Peptide mimetic drugs such as pep14-23, which mimics the N-terminus of DENV C protein, have been shown to block C interaction with lipid droplets (LDs) [27]. Because perturbing C–LD interaction has been shown to reduce virus production, a peptide-based drug that targets other functions of C is another promising way of reducing viremia.

Our study provides a structural understanding of C protein organization inside the immZIKV particle and illuminates its importance in the flavivirus assembly process. Our result may provide targets for designing drugs that could either prevent C protein from interacting with the RNA genome or the TM region of E and prM.

## Conclusion

The current cryoEM studies show that the C protein is important for virus assembly; its roles include (1) recruiting the RNA genome and (2) interacting with the transmembrane regions of prM–E, and also, (3) the presence of some f-C proteins may determine the spacing between the prM–E proteins, thus making the correct T = 3 infectious flaviviruses particle conformation. Taken together with the greater appreciation of C in the flavivirus assembly process, we hope that this will spur the development of therapeutics that target virus assembly.
